# Diagnostics of Torsade de Pointes in an oncohematology patient: A case report

**DOI:** 10.1002/ccr3.4292

**Published:** 2021-06-24

**Authors:** Ainur Bilmakhanbetova, Gulnur Zhakhina, Meruyert Beisenbay, Daulet Marat

**Affiliations:** ^1^ Center of Radiation and Functional Diagnostics National Research Oncology Center Nur‐Sultan Kazakhstan; ^2^ National Research Oncology Center Nur‐Sultan Kazakhstan; ^3^ Center Multidisciplinary Therapy National Research Oncology Center Nur‐Sultan Kazakhstan

**Keywords:** cardiotoxicity, Holter monitoring, oncohematology, sudden cardiac death, TdP‐Torsade de Pointes

## Abstract

Complex heart rhythm disturbances due to cardiotoxicity may not be identified in time, and this may have an unfavorable outcome for patients. Holter monitoring can be a solution to prevent sudden cardiac death of oncohematology patients.

## Graphical abstract

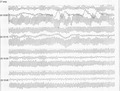

## 1 INTRODUCTION

The use of a multicomponent chemotherapy program in the treatment often leads to the development of cardiotoxicity.^1^ Due to side effects of chemotherapy, it can be temporarily interrupted and restarted after treatment of cardiovascular pathology or discontinue anticancer therapy. Currently, in clinical cardiology, the cardiotoxicity of drugs is a serious medical problem. Antiarrhythmic drugs, class IA III, antibacterial (macrolide and fluoroquinolone groups), antineoplastic drugs, cytostatics (Doxorubicin, Daunorubicin),^1^ some antidepressants, psychotropic and sedatives, anti‐fungal drugs (Amphotericin, Voriconazole (Vfend)), diuretic, and lipid‐lowering drugs are accounted as drugs that can lengthen the QT interval.^2^ Prolongation of the QT interval often appears as episodes of loss of consciousness and ventricular fibrillation, which causes cardiac arrest.^3^ Cardiovascular pathologies mostly occur among middle‐aged, elderly patients, and very rarely in young patients. In patients with no structural heart disease, sudden cardiac death (SCD) usually occurs due to the development of polymorphic ventricular tachycardia (VT) or VT of the Torsade de Pointes type.^4^

This paper presents a clinical case of acquired Long QT Syndrome (LQTS), which was caused by the use of an unfavorable combination of drugs. The condition of the cardiovascular system largely determines the survival rate of patients with oncohematological diseases in critical conditions. Identification of the acquired LQTS and its description is of great interest for the clinical practice of physicians from the point of view of the etiology of serious cardiac arrhythmias in patients without previous cardiovascular pathology.

## 2 CASE DESCRIPTION

Patient K., 20 year.old., was admitted to the oncohematological department of the National Research Oncology Center (NROC) in Kazakhstan, Nur‐Sultan from 12.10.2016 to 29.05.2017.

The disease manifestation started with weakness, dizziness, febrile temperature, shortness of breath, and swollen cervical lymph nodes in October 2016. The patient was diagnosed with acute lymphoblastic leukemia (option B III) based on laboratory tests, complete blood count, myelogram, bone marrow immunophenotyping, and molecular cytogenetic examination (FISH‐diagnostics). Before the disease, the patient had no health complaints.

According to the treatment protocol, the patient was fully examined before the start of chemotherapy.^5^ The patient underwent electrocardiography (ECG) and echocardiography. Electrocardiography result from 13.10.2016: sinus rhythm, heart rate of 86 beats per minute, and an electrical axis of the heart were normal. Echocardiogram from 13.10.2016: the chambers of the heart were not enlarged. The global, local contractile function of the myocardium was satisfactory, the pericardium was normal, and no structural pathology of the valvular apparatus of the heart was revealed.

According to the clinical protocol,^5^ five courses of chemotherapy were carried out, from 14.10.2016 to 29.05.2017, which included glucocorticosteroid (Dexamethasone), cytostatic drugs (Cyclofosmamide, Citrarbin, and Mercaptopurine), anthracycline antibiotics, and cytostatics (Doxorubicin and Daunorubicin).

Myelotoxic agranulocytosis was complicated with febrile neutropenia, gram‐positive sepsis (Staphylococcus aureus), and probable invasive pulmonary aspergillosis. The patient was additionally prescribed the following drugs: antibiotic Vancomycin, antimycotic therapy with Amphotericin, and Voriconazole (Vfend).

**FIGURE 1:**
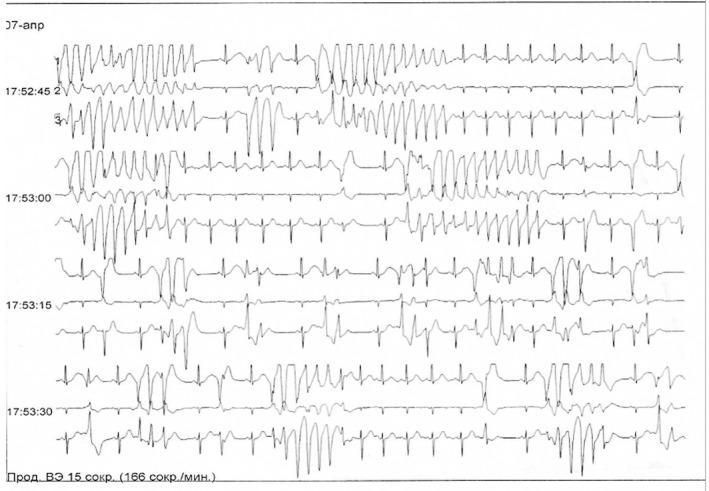
Ventricular tachycardia Torsade de Pointes

**FIGURE 2:**
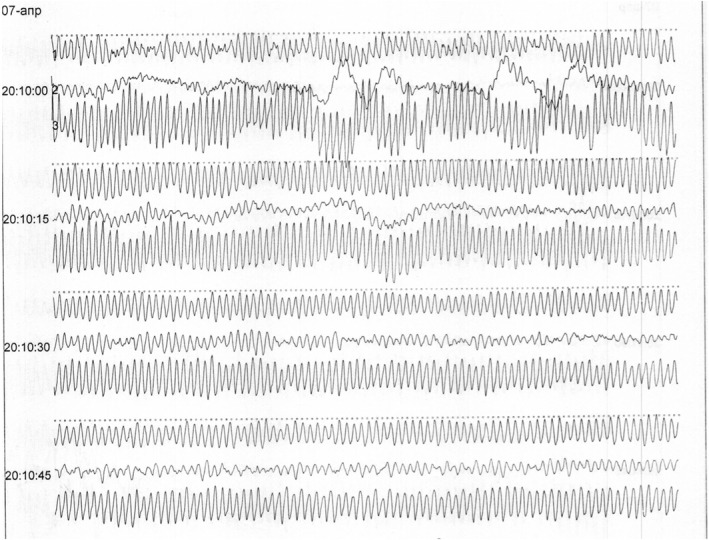
Ventricular fibrillation with a heart rate of 330 beats per minute. Sudden cardiac death of the patient

Laboratory tests were carried out during the entire treatment period, and blood biochemical tests and blood electrolytes were within normal limits. In the course of treatment, the patient underwent an ECG study dated April 5, 2017. The ECG showed frequent premature ventricular contraction (PVC) beats, an episode of sustained ventricular tachycardia with a ventricular rate of 166 beats per minute. The diagnosis was “Violation of the heart rhythm: Frequent premature ventricular contraction beats (paired), and episodes of ventricular tachycardia class 4B" according to Lown. The patient was additionally recommended for a Holter monitor. On April 7, 2017, in the evening, the patient lost consciousness and was transferred to the intensive care unit. The patient's condition deteriorated sharply, and there was a sudden cardiac death due to a disturbance in the rhythm of the heart, namely, ventricular tachycardia "pirouette" (Torsade de Pointes; Figure 1), which turned into ventricular fibrillation (Figure 2). In connection with ventricular fibrillation lasting about 9 minutes, cardiac resuscitation was immediately performed to restore the rhythm. Emergency cardioversion is recommended for life‐threatening arrhythmias according to the European indication class IC.^6^

During the incident, the patient was undergoing Holter monitoring, and the life‐threatening heart rhythm disturbance "Torsade de Pointes" (TdP) was detected. The patient underwent 24‐hour Holter monitoring.

**FIGURE 3:**
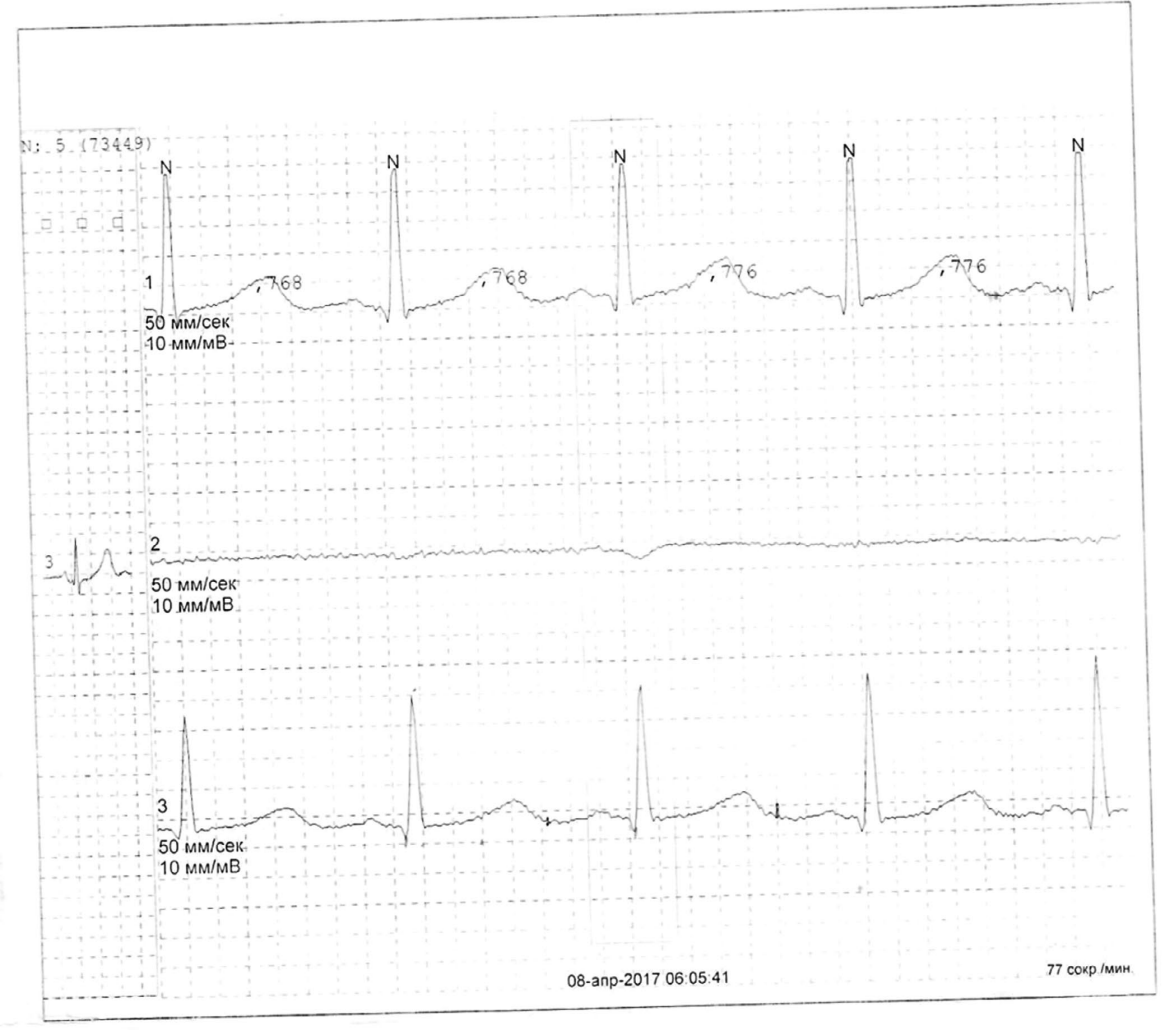
Prolongation of the QT/QTc interval of 500/560 msec

On the ECG, lengthening of the QTc interval was not detected; however, it was found on 24‐hour Holter monitoring (Figure 3). Prolongation of the corrected QTc interval is a predictor of cardiac arrest. Its lengthening against the background of pharmacotherapy in the patient manifested itself as cardiotoxicity and posed a great threat to the patient's life. The patient received antiarrhythmic therapy according to the Cordarone 600 mg/s scheme for 2 weeks, and then 400 mg/s for 2 weeks, then 200 mg/s for 3 months.

A month after the incident, a control 24‐hour Holter monitoring was performed, and ventricular and supraventricular activity was not detected. The QT interval with an average heart rate of 85 beats per minute was 400 msec, and QTc was 480 msec.

The patient underwent five courses of chemotherapy, and complete remission was achieved. The patient was discharged in satisfactory condition.

## 3 DISCUSSION

One of the most important and significant issues of cardiology is the early detection and treatment of patients with a high risk of sudden cardiac death (SCD). The most dangerous disease with the risk of developing SCD of arrhythmogenic genesis is long QT interval syndrome, in which the risk of developing SCD reaches 71%.^7^ According to a prospective study by the International LQTs Registry, 57% of SCD cases occur before 20.^8^ In 2016, the ESC Clinical Practice Guidelines Committee released a guideline regarding the treatment of cancer patients with chemoradiation therapy, as a result of which cardiovascular toxicity occurred.^9^ A large group of chemotherapeutic drugs has a cardiotoxic effect, which can be expressed as asymptomatic ECG changes and myocardial infarction, as well as the development of toxic cardiomyopathy with symptoms of severe heart failure.^8^, ^9^

Antineoplastic antibiotics of the anthracycline group and cytostatic drugs (Daunorubicin, Doxorubicin) have side effects on the cardiovascular system. Doxorubicin is one of the drugs that can cause acute or late forms of cardiotoxicity.^1^, ^10^ The consequence of its cardiotoxicity is an arrhythmia, which can develop at any time (Table 1).

**TABLE 1:** Diagnostics "Torsade de Pointes" using Holter monitor

Indicator	Result
Heart rhythm	Sinus rhythm, sinus arrhythmia.
Average heart rate	77 beats per min
Minimum heart rate	56 beats per min
Maximum heart rate	144 bears per min, sinus rhythm
Ventricular extrasystoles	A total of 3872:2242 single, ventricular arrhythmia, and 1630 episodes of ventricular tachycardia "Torsade de Pointes" (TdP) were detected per day (Figure 1)
Ventricular fibrillation	1 episode of ventricular fibrillation with a heart rate of 348 beats per minute lasting 9 minutes, from 20:09 to 20:18. (sudden cardiac death; Figure 2)
QT interval	With an average heart rate of 77 beats per minute, the QT interval was 500 msec and the corrected QTc was 560 msec. The lengthening of the QTc interval was transient (Figure 3)

Multicomponent therapy leads to multiple complications, which in turn require correction and treatment, as well as the appointment of additional medications. Thus, when prescribing medications, it is necessary to keep in mind that the risk of death of the patient and the development of "Torsade de Pointes" increase ^7^ ; therefore, it is preferable to perform Holter monitoring. The lengthening of this interval is often associated with drug‐induced cardiotoxicity.^8^ In this clinical case, lengthening of the QT/QTc interval was transient, and the patient had ventricular fibrillation. After thoughtful discussions, the drug of choice was Cordarone (generic name‐Amiodarone) to prevent relapses. However, cardiologists should keep in mind that we regularly monitored the QT/QTc interval while treating the patient with it. The patient was prescribed Amiodarone under the control of monitoring the QT interval to prevent sudden cardiac death. Ventricular tachycardia "Torsade de Pointes" (TdP) can clinically manifest as episodes of loss of consciousness and often end in ventricular fibrillation, which is the immediate cause of cardiac arrest.^11^

The importance of this clinical case is in the fact that it is not always possible to register or find ventricular fibrillation TdP, a life‐threatening heart rhythm disturbance that the patient had during the day, because the doctors had no suspicion of complex heart rhythm disturbances. In our clinical case, sudden cardiac death occurred due to the cardiotoxicity of drugs: the anthracycline group of antibiotics and anti‐flu drugs. We suppose that side effects and drug incompatibility have led to a prolongation of the QT/QTc interval. The QT interval after correction according to the Bazett formula is considered to be prolonged, with a duration of more than 450 msec in men and more than 470 msec in women.^8^ The value of the QT interval of more than 500 msec is a predictor of ventricular arrhythmias and cardiac arrest; therefore, it is recommended to immediately discontinue the drugs causing these changes.^7^ To date, there are a lot of studies that carefully describe the various causes of acquired prolongation of the QTc interval.^10^, ^11^, ^12^ But it is not always possible to notice a life‐threatening change in the heart rate; hence, it is advisable to prescribe daily ECG monitoring to observe the QTc interval for a more accurate and detailed diagnosis in oncohematological patients.

## 4 CONCLUSION

In this case report, a drug‐induced prolongation of the QT interval was identified, which led to the patient's sudden cardiac death. Patients who receive combinations of drugs that affect the duration of the QT interval should be warned about the need to promptly inform the attending physician about any symptoms that may be manifestations of Torsade de Pointes.

Hematologists‐oncologists must be fully aware of possible cardiac arrhythmias, and their close collaboration with cardiologists will lead to better cardiovascular risk stratification, monitoring, and treatment.

In order to detect asymptomatic prolongation of the QT interval of more than 500 msec, it is necessary to regularly perform an electrocardiographic examination. It is important to share cases of cardiac arrhythmias associated with a combination of different drugs to reduce mortality from arrhythmias in a cohort of oncohematology patients. This clinical case that showed more careful monitoring of the QT/QTc interval during the patient's treatment can have a beneficial effect on the successful outcome of the prescribed therapy.

## CONFLICT OF INTEREST

None declared.

## AUTHOR CONTRIBUTIONS

AB: performed a full analysis of data from Holter monitor, monitored the patient's health, and wrote this case report; DM: prescribed Holter monitoring based on ECG result and monitored the patient's health; MB: performed an echocardiogram of the heart's movement and monitored the patient's health; GZ: wrote this case report.

## ETHICAL APPROVAL

Written informed consent was obtained from the patient for publication of this case report and any accompanying images. This case report was conducted in compliance with the principles of the Declaration of Helsinki. The Ethical Committee of National Research Oncology Center (permit number №10) approved this study.

## Data Availability

The data that support the findings of this study are available from the corresponding author upon reasonable request.

## References

[1] Dazzi H , Kaufmann K , Follath F . Anthracycline‐induced acute cardiotoxicity in adults treated for leukaemia. Analysis of the clinico‐pathological aspects of documented acute anthracycline‐induced cardiotoxicity in patients treated for acute leukaemia at the University hospital of Zurich. Switzerland, between 1990 and 1996. Ann Oncol. 2001;12(7):963‐966. 11521803 10.1023/a:1011196910325

[2] Nousiainen T , Jantunen E , Vanninen E , et al. Acute neurohumoral and cardiovascular effects of idarubicin in leukemia patients. Eur J Haematol. 1998;61(5):347‐353. 9855251 10.1111/j.1600-0609.1998.tb01099.x

[3] Lee KW , Okin PM , Kligfield P , Stein KM , Lerman BB . Precordial QT dispersion and inducible ventricular tachycardia. Am Heart J. 1997;134(6):1005‐1013. 9424059 10.1016/s0002-8703(97)70019-x

[4] Zipes DP , Wellens HJ . Sudden cardiac death. Circulation. 1998;98:2334‐2351. 9826323 10.1161/01.cir.98.21.2334

[5] Afanasyev BV , Rakhimbekova GA , Pivovarova IA . Clinical protocol for diagnosis and treatment of acute lymphoblastic leukemia in adults. Protocol #6. Astana, Kazakhstan: Republican Center for Health Development of the Ministry of Health and Social Development of the Republic of Kazakhstan; 2015.

[6] Monsieurs KG , Nolan JP , Bossaert LL , et al. European resuscitation council guidelines for resuscitation 2015 Section 1. Executive summary. Resuscitation. 2015;95:1‐80. 26477410 10.1016/j.resuscitation.2015.07.038

[7] Day CP , McComb JM , Campbell RW . QT dispersion: an indication of arrhythmia risk in patients with long QT intervals. Heart. 1990;63(6):342‐344. 10.1136/hrt.63.6.342 PMC1024518 2375895

[8] Bovelli D , Plataniotis G , Roila F , et al. Cardiotoxicity of chemotherapeutic agents and radiotherapy‐related heart disease: ESMO clinical practice guidelines. Ann Oncol. 2010;21(suppl_5):v277‐v282. 20555097 10.1093/annonc/mdq200

[9] Zamorano JL , Lancellotti P , Rodriguez Muñoz D , et al. 2016 ESC position paper on cancer treatments and cardiovascular toxicity developed under the auspices of the ESC committee for practice guidelines: the task force for cancer treatments and cardiovascular toxicity of the European society of cardiology (ESC). Eur Heart J. 2016;37(36):2768‐2801. 27567406 10.1093/eurheartj/ehw211

[10] Shuykova KV , Storozhakov GI , Gendlin GE , et al. The Case of Acute Anthracyclines Cardiotoxicity. Presented at: Abstracts of XXXIII World Congress of the ISH. Jerusalem, Israel: 2010.

[11] Zipes DP , Camm AJ , Borggrefe M , et al. ACC/AHA/ESC 2006 guidelines for management of patients with ventricular arrhythmias and the prevention of sudden cardiac death—executive summary: a report of the American college of cardiology/American heart association task force and the European society of cardiology committee for practice guidelines (writing committee to develop guidelines for management of patients with ventricular arrhythmias and the prevention of sudden cardiac death). J Am Coll Cardiol. 2006;48(5):1064‐1108. 10.1016/j.jacc.2006.07.010 16949478

[12] Strevel EL , Ing DJ , Siu LL . Molecularly targeted oncology therapeutics and prolongation of the QT interval. J Clin Oncol. 2007;25(22):3362‐3371. 17664484 10.1200/JCO.2006.09.6925

